# Neurometabolite Changes After Transcranial Photobiomodulation in Major Depressive Disorder: A Randomized Controlled Trial Investigating Dose-Dependent Effects

**DOI:** 10.3390/jcm14103402

**Published:** 2025-05-13

**Authors:** David R. A. Coelho, Ümit Tural, Aura Maria Hurtado Puerto, Katherine Anne Collins, Maia Beth Gersten, Zamfira Parincu, Kari Siu, Dan Vlad Iosifescu, Eva-Maria Ratai, Paolo Cassano, Akila Weerasekera

**Affiliations:** 1 Division of Neuropsychiatry and Neuromodulation, Massachusetts General Hospital, Boston, MA 02129, USA; daraujocoelho@mgh.harvard.edu (D.R.A.C.);; 2 Department of Psychiatry, Harvard Medical School, Boston, MA 02114, USA; 3 Nathan Kline Institute for Psychiatric Research, Orangeburg, NY 10962, USA; 4 Department of Psychiatry, New York University Grossman School of Medicine, New York, NY 10016, USA; 5 Athinoula A. Martinos Center for Biomedical Imaging, Charlestown, MA 02129, USA; 6 Department of Radiology, Massachusetts General Hospital, Boston, MA 02114, USA; 7 Psychotic Disorders Division, McLean Hospital, Belmont, MA 02478, USA; 8 McLean Imaging Center, McLean Hospital, Belmont, MA 02478, USA

**Keywords:** transcranial photobiomodulation, photobiomodulation, major depressive disorder, neurometabolites, dose-dependent

## Abstract

**Background:** Transcranial photobiomodulation (t-PBM) is a promising non-invasive therapy for Major Depressive Disorder (MDD). MDD is associated with altered brain metabolism, including changes in N-acetylaspartate (NAA), choline (Cho), and creatine (Cr). This study assessed the effects of varying t-PBM doses on neurometabolite levels in the dorsolateral prefrontal cortex (dlPFC) and their correlations with clinical outcomes. **Methods:** In this randomized, sham-controlled, cross-over study, 33 adults with MDD received one session of t-PBM at low, medium, and high doses and a sham treatment. Proton magnetic resonance spectroscopy (1H-MRS) measured NAA, Cho, and Cr pre- and post-treatment. Clinical outcomes were assessed using the Montgomery–Åsberg Depression Rating Scale (MADRS) and the Symptoms of Depression Questionnaire (SDQ). Statistical analyses included paired *t*-tests or Wilcoxon signed-rank tests for neurometabolite changes, and linear mixed-effects regression models for t-PBM dose, neurometabolites, and time effects. **Results:** NAA levels increased significantly (7.52 ± 0.777 to 8.12 ± 1.05 mmol/L for one session; 7.36 ± 0.85 to 7.85 ± 0.68 mmol/L across all sessions); however, these changes were not associated with specific t-PBM doses or sham. No significant changes were observed for Cho and Cr levels. Positive correlations were found between Cho levels and MADRS scores (r = 0.59, *p* = 0.017), and negative correlations between Cr levels and SDQ scores at the medium dose (r = −0.91, *p* = 0.011). **Conclusions:** While NAA levels increased, and correlations between neurometabolites and clinical outcomes were observed, these findings do not suggest a specific effect of t-PBM. Larger randomized controlled trials with optimized dosing protocols, extended follow-up, and advanced spectroscopy are needed to clarify the neurometabolic therapeutic potential of t-PBM in MDD.

## 1. Introduction

Major Depressive Disorder (MDD) is a serious mental health condition characterized by persistent sadness, hopelessness, and diminished interest or pleasure in activities, often impairing daily functioning [1]. Its complex causes involve genetic, biological, environmental, and psychological factors [2]. While psychotherapy and antidepressants are standard treatments [3], transcranial photobiomodulation (t-PBM) has emerged as a promising non-invasive therapy [4]. t-PBM delivers near-infrared (NIR) light to the brain, modulating neurobiological processes at the cellular level [5], and has been investigated for neuropsychiatric conditions such as MDD, bipolar disorder, Alzheimer’s and Parkinson’s diseases, neurodevelopmental disorders, Down syndrome, and traumatic brain injury [6,7,8,9,10,11,12,13,14,15,16]. Early clinical trials suggest t-PBM may be a safe and effective treatment for MDD, particularly for individuals resistant to conventional therapies [17,18,19]. However, despite its potential, the impact of t-PBM on neurometabolites, particularly its dose-dependent effects, remains underexplored.

Proton magnetic resonance spectroscopy (1H-MRS), a non-invasive imaging technique, provides insights into the biochemical changes associated with MDD and t-PBM therapy [20]. By measuring brain metabolites such as N-acetylaspartate (NAA), a marker of neuronal viability [21]; creatine (Cr), a marker of cellular energy metabolism [22]; choline (Cho), a marker of cell membrane turn-over [23]; and myo-inositol (mIns), a glial marker [24], 1H-MRS can be used to assess the effects of t-PBM on neurometabolites in brain regions implicated in MDD, such as the prefrontal cortex [25,26]. Studies combining t-PBM and 1H-MRS may enhance understanding of the metabolic effects of t-PBM and its efficacy as an evidence-based intervention for MDD.

This study aimed to (1) evaluate the effects of varying doses of t-PBM on neurometabolite levels (NAA, Cho, Cr and mIns) in the dorsolateral prefrontal cortex (dlPFC) of patients with MDD and (2) to investigate the correlation between these changes in neurometabolite levels and changes in depression severity.

## 2. Materials and Methods

### 2.1. Study Design

This study was a randomized, single-blinded, sham-controlled, cross-over design. It was part of a larger initiative funded by the National Institute of Mental Health (NIMH) (ClinicalTrials.gov identifier: NCT04366258). The full methodology was previously outlined in an established protocol and detailed in a prior publication [19,27]. The study was conducted across three primary centers: Massachusetts General Hospital (MGH), New York University (NYU), and the Nathan Kline Institute for Psychiatric Research (NKI). Ethical approval was obtained from the Institutional Review Board at NYU (IRB number: s20-00217).

### 2.2. Randomization

Participants were randomized to a specific sequence of the four t-PBM/sham doses using a computer-generated schedule. A total of 24 possible sequence orders existed; from these, 8 orders (derived from two distinct 4 × 4 Latin squares) were selected for use at each site. The study statistician provided each site with a block-randomized list assigning the order in which participants received the four conditions. This cross-over design ensured that all participants experienced each dose condition and served as their own control.

### 2.3. Blinding

The study was conducted under single-blinded conditions, where participants were unaware of their assigned intervention. For the sham condition, the t-PBM device was left on standby, such that the device appeared activated, with a visible light, but no near-infrared stimulation was delivered. In addition, at each visit, participants completed a Perceived Blinding Questionnaire to assess their belief about the treatment received, the strength of that belief, and their expectation of benefit. Outcome assessors, however, were not blinded to the order of treatments.

### 2.4. Eligibility Criteria

Participants were eligible for inclusion if they were aged between 18 and 65 years, met the criteria for MDD as described in the Diagnostic and Statistical Manual of Mental Disorders, Fifth Edition (DSM-5), and were not classified as treatment-resistant (defined as failure to respond to no more than two antidepressant treatments during the current episode). Additionally, participants had to be either unmedicated or on stable doses of antidepressants. Exclusion criteria included significant suicidal risk, a history of psychotic or bipolar disorders, substantial cognitive impairment, neurological disorders, traumatic brain injury, pregnancy or breastfeeding, and non-adherence to treatment protocols.

### 2.5. Intervention

All subjects underwent four t-PBM dosing conditions in randomized order: low, medium, high, and sham, with each session spaced approximately one week apart. These doses refer to specific, quantitatively defined irradiance and energy parameters, as demonstrated in Table 1. Session durations varied by dose: low (20 min), medium (6 min), high (10 min), and sham (10 min). The target area was the dlPFC bilaterally (F3 and F4 EEG electrode locations). The t-PBM device used in this study was the tPBM-2.0, an investigational modification of LiteCure’s LightForce^®^ EXPi Deep Tissue Laser Therapy™ System, manufactured and supplied by LiteCure LLC, New Castle, DE, USA. The device delivers NIR laser light at a wavelength of 808 nm, configured to administer different irradiance levels and emission modes based on randomized treatment conditions. The laser system uses a flexible, double-sheathed optical fiber connected to a helmet designed for secure placement on the scalp, ensuring precise NIR delivery to the target regions.

### 2.6. Protocol Adherence and Site Standardization

The intervention was delivered inside the magnetic resonance imaging (MRI) scanner for all participants, in accordance with the study design. Subjects entered the scanner head-first in a supine position, wearing the MRI-compatible headset and laser safety glasses. Stimulation was administered with lights turned off in the scanner room to maintain consistency across sessions. To ensure procedural consistency, weekly meetings were held with staff across MGH, NYU, and NKI to review study protocols and address any procedural questions. Staff at each site received similar overarching training and operated under a shared standard operating procedure, including device calibration, session setup, and safety protocols.

### 2.7. Outcomes

Imaging was conducted using a 3T Siemens Trio MRI scanner (Siemens Healthineers, Erlangen, Germany) with 12-channel transmit/receive head coils. T1-weighted MPRAGE sequences were used for anatomical localization, voxel placement, and partial volume correction of cerebrospinal fluid. Single-voxel proton magnetic resonance spectroscopy (1H-MRS) data were collected pre- and post-treatment, approximately 15 min after t-PBM sessions. A voxel (30 × 30 × 15 mm^3^) was placed in the left frontal lobe for magnetic resonance spectroscopy (MRS) acquisition using a Point RESolved Spectroscopy (PRESS) sequence [28] (TR = 2000 ms, TE = 30 ms, bandwidth = 2000 Hz). The acquisition included 128 signal averages, with a total acquisition time of 4 min and 48 s for the main spectroscopy sequence and 16 s for the water-suppressed spectroscopy sequence (four signal averages, acquisition duration = 514 ms).

**Figure 1:**
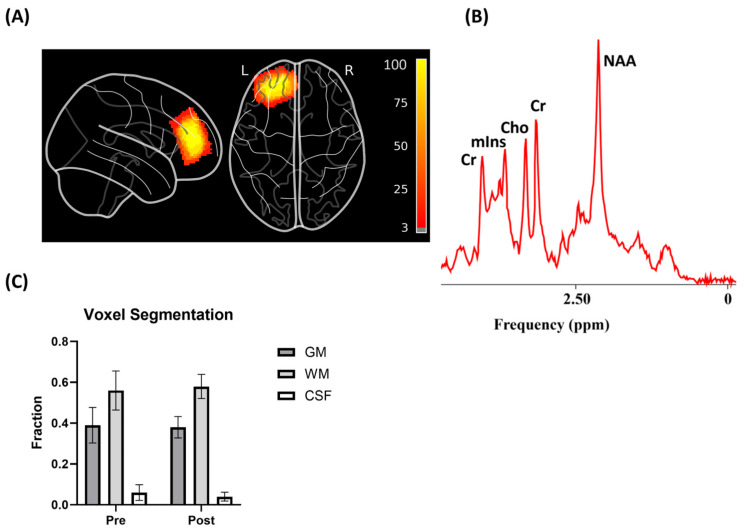
Magnetic resonance spectroscopy (1H-MRS) voxel placement, segmentation, and magnetic resonance (MR) spectra. (**A**) Both pre- and post- left prefrontal MRS voxel (13 cm^3^) overlap density maps for all participants in sagittal and axial planes. Individual MRS voxels were transformed to the MNI space and then combined to show the overlap. (**B**) Representative spectrum from the left dorsolateral prefrontal cortex, showing prominent peaks for N-acetylaspartate (NAA), creatine (Cr), choline (Cho), and myo-inositol (mIns). (**C**) Voxel composition of gray matter (GM), white matter (WM), and cerebrospinal fluid (CSF) in pre- and post-treatment scans.

Neurometabolites measured included total NAA (the sum of N-acetylaspartate [NAA] and N-acetylaspartylglutamate [NAAG]), total Cho (phosphorylcholine [PCho] and free choline), and total Cr (creatine and phosphocreatine). Quantification was performed using the jMRUI toolkit v6.01 and the QUEST algorithm, with phase correction and water-referenced spectra. Only metabolites with a Cramer–Rao lower bound (CRLB) < 25% were included. Tissue segmentation using Gannet and SPM12 corrected for partial volume effects in gray matter, white matter, and cerebrospinal fluid (CSF). Only metabolites with a Cramer–Rao lower bound (CRLB) < 25% were considered for further analysis. The levels were corrected for partial volume effects by registering the MRS voxel to the T1 anatomical space and segmenting it into gray matter, white matter, and CSF using the Gannet toolkit v3.13 [29] and SPM12 [30]. The segmented tissue fractions were applied to partial volume corrected for the metabolite levels using jMRUI-QUEST to account for CSF content, as outlined in the literature [31]. The T1 and T2 values for gray matter, white matter, and CSF used in this study were 1331, 832, and 3817 ms and 110, 79, and 503 ms, respectively. The metabolite relaxation times used for calculating final corrected levels were based on previous studies [32,33]. Figure 1 illustrates the MRS voxel placement, segmentation, and a representative spectrum.

To assess clinical outcomes, we applied the Montgomery–Åsberg Depression Rating Scale (MADRS) [34] and the Symptoms of Depression Questionnaire (SDQ) [35]. MADRS scores were used to evaluate changes in depressive symptoms, while SDQ scores captured a broader range of depression-related symptoms, including anxiety.

### 2.8. Study Data Analysis Scheme

**Figure 2:**
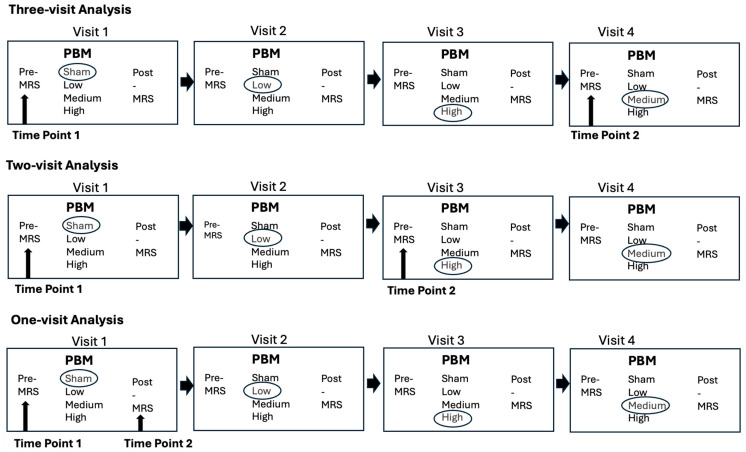
Study design and magnetic resonance spectroscopy (1H-MRS) data analysis schemes. This figure illustrates the three MRS data analysis schemes used in this study. In the three-visit analysis, participants received sham, low, medium, or high transcranial photobiomodulation (t-PBM) doses across three visits, with time point 1 (pre-MRS) at the start of visit 1 and time point 2 (pre-MRS) at the start of visit 4. The two-visit analysis involved participants receiving sham, low, medium, or high t-PBM doses across two visits, with time point 1 (pre-MRS) at the start of visit 1 and time point 2 (pre-MRS) at the start of visit 3. In the one-visit analysis, participants received a single dose (sham, low, medium, or high t-PBM) during a single visit, with time point 1 (pre-MRS) at the start and time point 2 (post-MRS) at the end of visit 1.

Each participant in this study underwent three randomized t-PBM treatments and one sham session, with each session spaced one week apart. Pre- and post-treatment MRI/MRS data were collected during all sessions. Three separate analyses were conducted to assess changes in neurometabolite levels following t-PBM treatment (Figure 2). Due to the poor quality of most post-treatment MRS spectra, only pre-treatment MRS data were included in the three-visit and two-visit analyses. This limitation prevented the inclusion of the final t-PBM session in these analyses. For the one-visit analysis, only high-quality post-treatment MRS spectra were utilized.

### 2.9. Statistical Analysis

Sociodemographic and baseline clinical characteristics were reported as frequencies, percent, or mean (±SD), depending on the features of the variables. We applied paired *t*-test or Wilcoxon signed rank test to compare variables, depending on their distributional characteristics. Linear mixed-effects regression models with restricted maximum likelihood algorithms were fitted in R software version 4.4.1 [36] using the “lme4” package [37]. The models in the prediction of MADRS and SDQ scores included sex, age, levels of neurometabolites, time (pre-, post-), and NIR doses as fixed factors and repeated measurements across participants as the random factor. The violations and robustness of the models were evaluated with residual histograms, normality test of residuals, residual vs. fitted plots, and QQ plots. A *p*-value of <0.05 was used to denote statistical significance. The Benjamini–Hochberg *p* adjustment method was used to control false discovery rate when multiple tests were performed. When a statistically significant difference was found, the effect size (Cohen’s *d*) was calculated and presented.

## 3. Results

**Figure 3:**
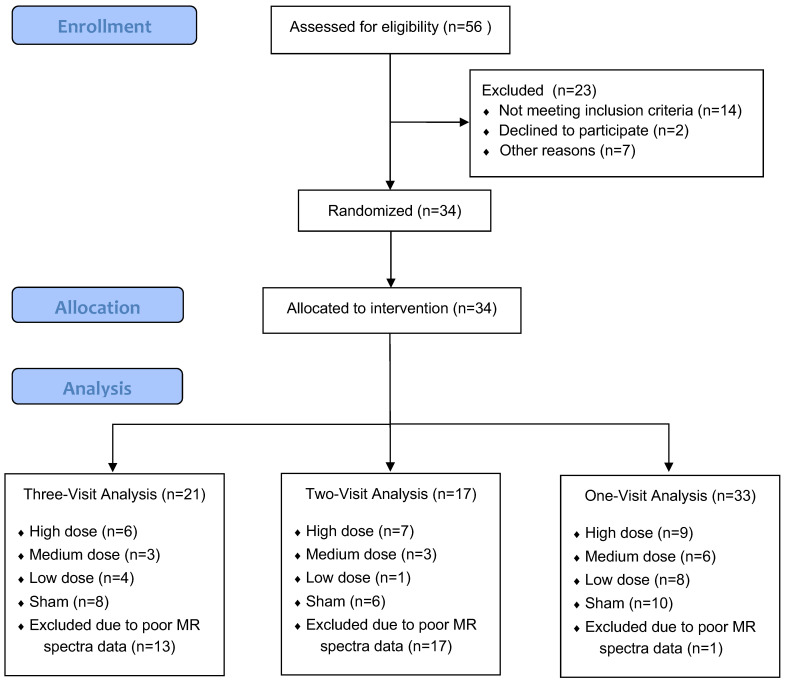
Participant flow diagram for MR spectra dataset inclusion across analysis schemes. This flowchart depicts participant enrollment, allocation, and inclusion in each analysis scheme (one-visit, two-visit, and three-visit) based on valid MRS data. A total of 56 individuals were assessed for eligibility, with 34 randomized to intervention. Final sample sizes varied by analysis due to exclusion of visits with poor MR spectra quality.

All MR spectra used in the analysis had a signal-to-noise ratio (SNR) >15 and a line width of the unsuppressed water signal at half height (FWHM) of <13 Hz (9 ± 3 Hz). The Cramer–Rao lower bounds for NAA, Cr, and Cho were below 7%. However, the majority of quantified mIns signals exhibited CRLBs exceeding 25% and were therefore excluded from the analysis. The number of valid spectra per dosing condition (high, medium, low, sham) is detailed in the corresponding analysis subsections below and in Figure 3. Neither age nor gender was significantly associated with the main outcomes in the statistical analyses of metabolite levels, MADRS, or SDQ at any visit frequency.

### 3.1. Three-Visit Analysis

#### 3.1.1. Demographics

A total of 21 (15 female and 6 male) patients with MDD were included in this analysis. The mean age of the sample was 37.5 ± 15. The mean baseline MADRS score was 26.8 ± 6, and at the end of the study was 20.8 ± 10. The mean baseline SDQ score was 131.7 ± 25, and at the end of the study was 117.9 ± 25. Participants received t-PBM doses or sham as follows: high dose: n = 6; medium dose: n = 3; low dose: n = 4; sham: n = 8.

#### 3.1.2. Changes in Neurometabolite Levels

**Figure 4:**
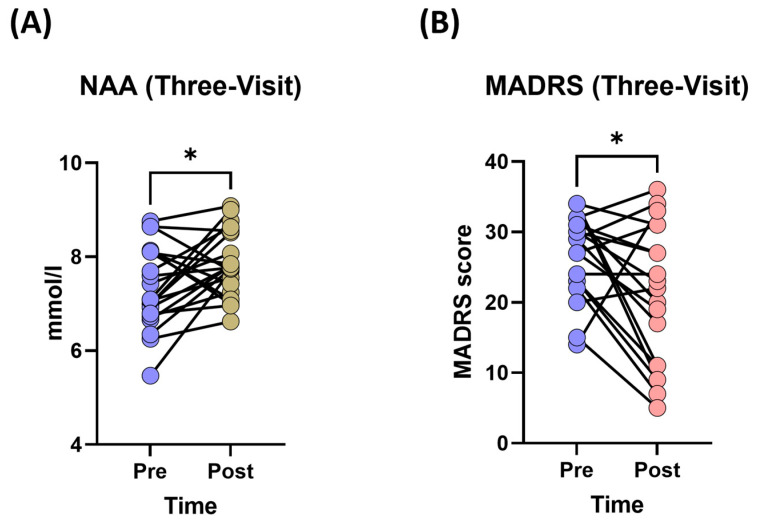
N-acetylaspartate (NAA) levels and Montgomery–Åsberg Depression Rating Scale (MADRS) scores. (**A**) Changes in NAA levels (pre- and post-treatment) following three sessions of t-PBM. (**B**) Changes in MADRS scores (pre- and post-treatment) following three sessions of t-PBM. Data are presented as individual changes; * *p* < 0.05.

After three visits, patients’ dlPFC NAA levels were significantly different between pre- and post- treatment; specifically, the mean levels increased (7.36 ± 0.85 to 7.85 ± 0.68 mmol/L, Cohen’s *d* = 0.64) after t-PBM treatment (F_(1,20)_ = 5.37, *p* = 0.031) (Figure 4A). However, this NAA increase was not associated with any t-PBM doses or sham (F_(3,15)_ = 0.65, *p* > 0.184). Furthermore, there were no significant changes in Cho (F_(1,20)_ = 0.001, *p* = 0.973) or Cr levels (F_(1,20)_ = 2.50, *p* = 0.129) post-treatment compared to pre-treatment.

#### 3.1.3. Changes in Depression Severity (MADRS Scores)

We observed a significant decrease in the mean MADRS scores following t-PBM treatment. The linear mixed-effects model showed that there was a main effect of time (pre/post) (F_(1,18)_ = 6.18, *p* = 0.023) on the MADRS scores (Figure 4B). However, the MADRS score change was not significantly different among t-PBM doses and sham (F_(3,13)_ = 0.14, *p* = 0.922). We also included neurometabolite levels in this model to assess their effect on the MADRS score; however, none were found to be significant (minimum *p* > 0.176).

#### 3.1.4. Changes in Depression Severity (SDQ Scores)

The SDQ scores were not significantly different pre- and post- treatment. However, a trend towards decreasing SDQ scores post-treatment compared to pre-treatment was observed (F_(1,16)_ = 4.12, *p* = 0.059). No significant association of t-PBM dose or neurometabolite levels on the SDQ scores was found (minimum *p* > 0.615).

### 3.2. Two-Visit Analysis

#### 3.2.1. Demographics

A total of 17 (13 female and 4 male) patients with MDD were included in this analysis. The mean age of the sample was 38.0 ± 14.5. The mean baseline MADRS score was 27.4 ± 6.30, and at the end of the study was 22.9 ± 8.81. The mean baseline SDQ score was 133 ± 27.6, and at the end of the study was 120 ± 21.3. Participants received t-PBM doses or sham as follows: high dose: n = 7; medium dose: n = 3; low dose: n = 1; sham: n = 6.

#### 3.2.2. Changes in Neurometabolite Levels

Since there were no participants in the sham arm at baseline and only one participant in the low dose arm at the endpoint, comparisons in the levels of neurometabolites were available for the high and medium t-PBM doses only. There was no significant change in NAA, Cho, and Cr levels following high and medium t-PBM doses. NAA (F_(2,24)_ = 1.27, *p* = 0.300), Cho (F_(2,25)_ = 1.16, *p* = 0.329), and Cr (F_(2,22)_ = 0.40, *p* = 0.675) levels of neurometabolites were not associated with treatment.

#### 3.2.3. Changes in Depression Severity (MADRS Scores)

**Figure 5:**
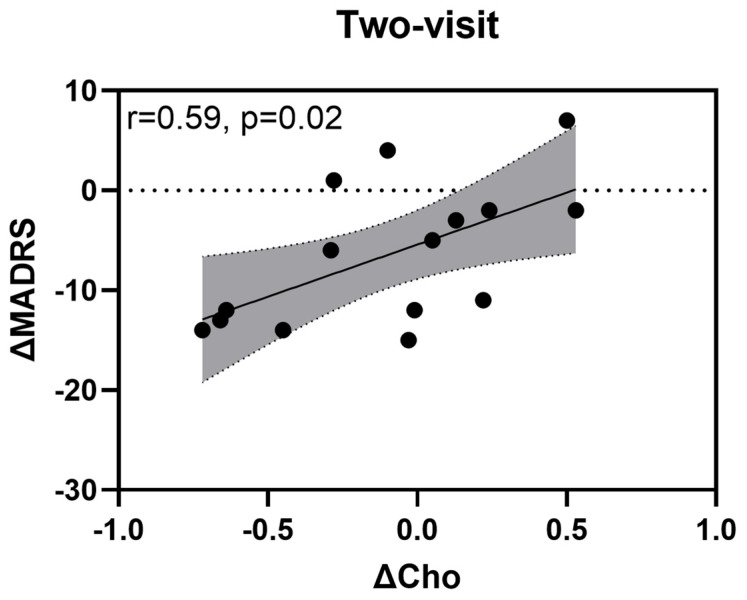
Correlation between choline (ΔCho) levels and Montgomery–Åsberg Depression Rating Scale (ΔMADRS). The scatter plot shows the relationship between changes in choline levels (ΔCho) and changes in MADRS scores (ΔMADRS) after two sessions of t-PBM. The dotted line represents no change in MADRS scores (ΔMADRS = 0). The grey area represents the 95% confidence interval for the estimated mean response. A significant positive correlation (r = 0.59, *p* = 0.02) was observed between ΔCho levels and ΔMADRS scores.

The linear mixed-effects model showed no main effect of t-PBM dose (F_(3,11.80)_ = 3.22, *p* = 0.062) or time (F_(1,10.57)_ = 0.11, *p* = 0.744) and no treatment × time interaction effect on MADRS scores (F_(2,17.29)_ = 1.99, *p* = 0.167). However, Cho levels significantly and positively impacted MADRS scores ($\hat{\beta }$ = 11.77, t = 2.70, df = 14.21, *p* = 0.017). A 1-unit increase in Cho level was associated with a 11.77-unit increase in MADRS score. The correlation between delta (post-pre) Cho levels and MADRS scores is summarized in Figure 5.

#### 3.2.4. Changes in Depression Severity (SDQ Scores)

No main effect of t-PBM dose (F_(3,12.51)_ = 1.96, *p* = 0.172) or time (pre/post) (F_(1,10.66)_ = 1.03, *p* = 0.333) on SDQ scores was observed. In addition, no treatment × time interaction effect on SDQ scores was observed (F_(2,20.30)_ = 3.05, *p* = 0.069). None of the levels of neurometabolites were significantly associated with SDQ score.

### 3.3. One-Visit Analysis

#### 3.3.1. Demographics

A total of 33 (27 female and 6 male) patients with MDD were included in this analysis. The mean age of the sample was 34.8 ± 14.6. The mean baseline MADRS score was 24.2 ± 8.07, and at the end of the study was 21.0 ± 8.76. The mean baseline SDQ score was 119 ± 25.9, and at the end of the study was 114 ± 24.3. Participants received t-PBM doses or sham as follows: high dose: n = 9; medium dose: n = 6; low dose: n = 8; sham: n = 10.

#### 3.3.2. Changes in Neurometabolites

**Figure 6:**
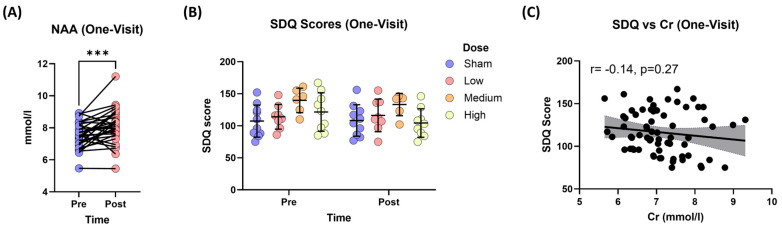
N-acetylaspartate (NAA) levels and Symptoms of Depression Questionnaire (SDQ) scores, and the correlation between creatine (Cr) levels and SDQ scores. (**A**) Changes in NAA levels (pre- and post-treatment) following one session of t-PBM. Data are presented as individual changes; *** *p* < 0.01. (**B**) Changes in SDQ scores (pre- and post-treatment) following one session of t-PBM. (**C**) The scatter plot shows the relationship between changes in Cr levels and changes in SDQ scores after one session of t-PBM. The grey area represents the 95% confidence interval for the estimated mean response. A significant negative correlation (r = −0.14, *p* = 0.027) was observed between Cr levels and SDQ scores for all doses.

NAA levels significantly increased from pre-treatment (7.52 ± 0.777 mmol/L) to post–t-PBM treatment (8.12 ± 1.05 mmol/L, t = 3.74, df = 32, *p* = 0.0007, Cohen’s *d* = 0.65) (Figure 6A). However, the increase in NAA levels was not significantly different between the t-PBM doses and sham (F_(3,43)_ = 0.696, *p* = 0.559). Choline and Cr levels were not significantly different in the post-treatment period compared to pre-t-PBM.

#### 3.3.3. Changes in Depression Severity (MADRS Scores)

The linear mixed-effects regression model showed that there were no significant main effects of treatment and time, and no treatment × time interaction effect on MADRS scores. Furthermore, none of the levels of neurometabolites were significantly associated with changes in MADRS scores.

#### 3.3.4. Changes in Depression Severity (SDQ Scores)

**Figure 7:**
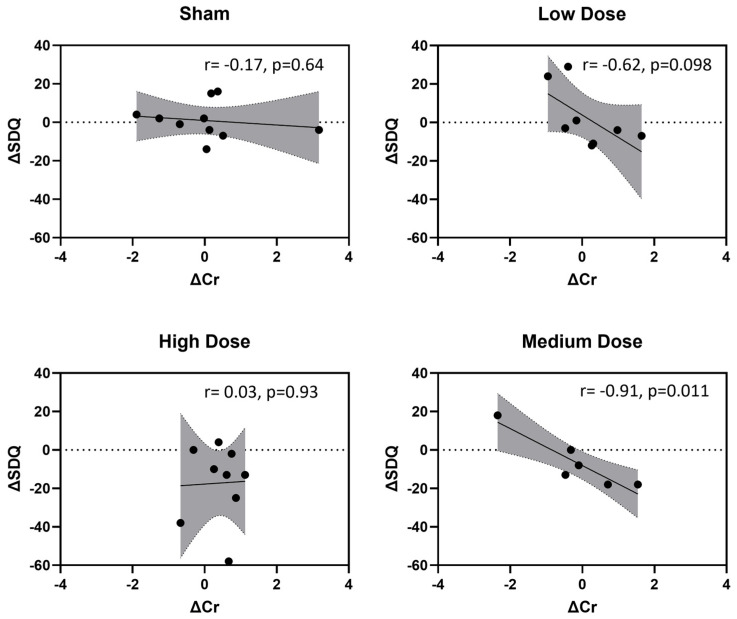
Correlation between changes in creatine (ΔCr) levels and the Symptoms of Depression Questionnaire (ΔSDQ) across transcranial photobiomodulation (t-PBM) treatment doses. The scatter plots show the relationship between changes in creatine levels (ΔCr) and SDQ scores (ΔSDQ) for each dose after one session of t-PBM. The dotted line represents no change in SDQ scores (ΔSDQ = 0). The grey area represents the 95% confidence interval for the estimated mean response. A significant and negative correlation (r = −0.91, *p* = 0.011) was observed between ΔCr levels and ΔSDQ scores at the medium t-PBM dose.

There was a significant main effect of t-PBM treatment on SDQ scores (F_(3,42.771)_ = 3.6385, *p* = 0.021, Cohen’s *d* = −0.31). However, there was no main effect of time (pre/post) or treatment × time interaction on SDQ scores (Figure 6B). Cr levels were significant and negatively impacted SDQ scores. A 1-unit increase in Cr level was associated with a 5.37-unit decrease in SDQ ($\hat{\beta }$ = −5.37, t = −2.29, df = 42.25, *p* = 0.027) (Figure 6C). Further analysis revealed that the medium dose was associated with a significant negative correlation between SDQ and Cr levels (Figure 7).

## 4. Discussion

Our study did not support a dose-dependent relationship between t-PBM and changes in neurometabolite levels. While an increase in NAA levels was observed, this finding was independent of the dose (irradiance level) of t-PBM therapy. There were correlations between neurometabolites and psychometric measures, including MADRS and SDQ scores, suggesting potential neurometabolic changes associated with MDD severity. However, it is important to note that these correlations do not establish causality and may reflect broader physiological or temporal fluctuations unrelated to specific treatment effects.

The increase in NAA levels in both the one-visit and three-visit analyses warrants further investigation. NAA, a marker of neuronal health and mitochondrial function, is synthesized by the mitochondrial enzyme aspartate N-acetyltransferase, directly linking its levels to mitochondrial metabolism [38]. Reduced NAA levels have been frequently reported in patients with MDD, reflecting impaired mitochondrial function and neuronal integrity [39]. Although t-PBM is hypothesized to enhance mitochondrial function by interacting with cytochrome c oxidase, thereby boosting adenosine triphosphate (ATP) production and reducing oxidative stress [5], the increase in NAA levels after a single t-PBM session was not statistically significantly associated with any of the t-PBM doses or sham and could be attributable to natural fluctuations or time-related changes. In contrast, other neuromodulatory interventions, such as repetitive transcranial magnetic stimulation (rTMS), have shown dose-dependent increases in NAA levels within the dlPFC after multiple sessions, which were associated with improvements in depressive symptoms [40]. Similarly, increases in NAA have been reported in a study investigating repeated t-PBM administration for chronic traumatic encephalopathy [41]. However, that study targeted the anterior cingulate cortex (ACC) rather than the dlPFC and employed different parameters, including longer session durations (22–40 min), higher treatment frequency (three times per week over six weeks), and NIR wavelengths ranging from 810 to 870 nm [41]. These differences in stimulation protocols and target regions could explain the discrepancies in findings, as the full biological and clinical effects of t-PBM may require repeated administration over several weeks.

The correlation between Cho levels and depression severity outcomes may offer insights into the pathophysiology of MDD, even though the observed changes in Cho levels were not dependent on t-PBM dose. While overall Cho levels remained stable post-therapy, we found a significant positive association between changes in Cho levels and changes in MADRS scores in the two-visit analysis (r = 0.59, *p* = 0.02). This finding suggests that increases in Cho levels compared to baseline were associated with less improvement in depressive symptoms, as higher MADRS scores indicate greater severity [34]. Brain Cho levels are considered markers of phospholipid synthesis and degradation, reflecting cell membrane turnover and integrity [42]. Alterations in choline metabolism have been implicated in psychiatric conditions, including MDD [43]. Our findings are consistent with prior work, such as Yang et al. (2016), who observed a positive association between Cho levels in the left dlPFC of adolescents with MDD and Beck Depression Inventory scores, indicating that Cho levels rise with the severity of MDD symptoms [44]. However, readers should be aware that this is a correlation analysis, and it should not be interpreted as a cause-and-effect relationship.

We observed a significant negative correlation between changes in Cr levels and changes in SDQ scores at medium t-PBM dose in the one-visit analysis (r = −0.91, *p* = 0.011), suggesting that higher Cr levels were associated with improved depression outcomes. However, it is important to emphasize that this association does not establish causality, as other factors may contribute to these observed changes. While overall Cr levels remained stable post-treatment, this dose-specific correlation may highlight the potential relevance of medium-dose t-PBM in modulating Cr-related pathways. Cr is a key regulator of ATP production, essential for maintaining cellular energy metabolism, neuronal activity, and synaptic plasticity [45,46]. Our findings align with prior research indicating that lower Cr levels in the prefrontal cortex are associated with greater depression severity, while higher Cr levels correlate with improved structural and functional outcomes, such as increased gray matter volume in the right superior medial frontal gyrus [47]. Our findings contribute to this growing evidence by indicating that Cr has the potential to serve as a biomarker for functional improvements in MDD, particularly in the context of medium-dose t-PBM. Interestingly, although no dose resulted in significant brain temperature elevations, the medium t-PBM dose demonstrated the lowest brain temperature fluctuations (σ^2^ = 0.29) compared to the low dose (σ^2^ = 0.47) and high dose (σ^2^ = 0.67) in a previous study [19]. Further research with larger sample sizes is needed to confirm the potential relationship between medium-dose t-PBM, Cr metabolism, and SDQ improvements.

Several limitations of this study should be acknowledged. While the cross-over design helps reduce inter-subject variability, the one-week interval between sessions may have introduced carryover effects that could influence neurometabolite outcomes. The limited small sample size, further reduced by excluding poor-quality data, limits generalizability and statistical power, complicating the detection of dose-dependent changes. Furthermore, some experts have argued that using the quantitative difference of geometric means may better accommodate the multiplicative, log-normal distribution of neurometabolic data [48]; however, we elected to test our hypotheses using traditional statistical methods given the exploratory nature of our study, and as a result we may have overlooked comparisons that alternative analyses could have identified as significant. The unbalanced number of participants across groups also adds variability, hindering dose-specific interpretations. Moreover, outcome assessors were not blinded to treatment order, introducing potential observer bias. Another limitation is the lack of phosphorus spectroscopy, which would have provided more detailed data on phospholipid metabolism; as such, we were only able to measure total Cho and total Cr. Lastly, since participants received only one session per dose of t-PBM, the study could not assess repeated treatment effects, and its short duration may not have captured gradual neurometabolic changes over a longer treatment period.

## 5. Conclusions

In conclusion, our study did provide evidence for a dose-dependent relationship between single-dose t-PBM administration and changes in neurometabolite levels. While an increase in NAA levels was observed, this change was not specific to t-PBM dosing and could be likely related to natural fluctuations or temporal effects of MDD. Correlations between neurometabolites, such as Cho and Cr, and psychometric measures, including MADRS and SDQ scores, indicate potential neurometabolic changes associated with MDD, though no causal relationships can be inferred given the study design. Future studies should prioritize larger, well-powered randomized controlled trials with refined dosing protocols, repeated dosing, sham-only control groups, and extended follow-up periods to assess long-term effects. Incorporating advanced spectroscopy techniques, such as phosphorus spectroscopy, and enhanced imaging protocols will be essential for reliable neurometabolite analysis and to further elucidate the therapeutic potential of t-PBM in MDD.

## Figures and Tables

**Table 1:** Transcranial photobiomodulation dose parameters.

Parameters	Low Dose	Medium Dose	High Dose	Sham
NIR Source	Laser	Laser	Laser	N/A
Wavelength (nm)	808	808	808	N/A
Area of Exposure (cm^2^)	24	24	24	24
Peak Irradiance (mW/cm^2^)	~50	~300	~850	0
Average Irradiance (mW/cm^2^)	~50	~300	~300	0
Exposure Time (s)	1200	333	600	0
Average Fluence (J/cm^2^)	~60	~100	~180	0
Total Energy (kJ)	~1.4	~2.4	~4.3	0
Wave Mode	Continuous	Continuous	Pulsed	N/A
Duty Cycle (%)	N/A	N/A	33	N/A
Pulse Rate (Hz)	N/A	N/A	~40	N/A

N/A: Not applicable.

## Data Availability

The data presented in this study are available on request from the corresponding author.

## References

[1] Otte C., Gold S.M., Penninx B.W., Pariante C.M., Etkin A., Fava M., Mohr D.C., Schatzberg A.F. (2016). Major Depressive Disorder. Nat. Rev. Dis. Primers.

[2] Alberti A., Araujo Coelho D.R., Vieira W.F., Moehlecke Iser B., Lampert R.M.F., Traebert E., da Silva B.B., de Oliveira B.H., Leão G.M., de Souza G. (2024). Factors Associated with the Development of Depression and the Influence of Obesity on Depressive Disorders: A Narrative Review. Biomedicines.

[3] Wang Z., Ma X., Xiao C. (2019). Standardized Treatment Strategy for Depressive Disorder. Adv. Exp. Med. Biol..

[4] Hamblin M.R. (2016). Shining Light on the Head: Photobiomodulation for Brain Disorders. BBA Clin..

[5] Hamblin M.R. (2017). Mechanisms and Applications of the Anti-Inflammatory Effects of Photobiomodulation. AIMS Biophys..

[6] Vieira W.F., Iosifescu D.V., McEachern K.M., Gersten M., Cassano P. (2023). Photobiomodulation: An Emerging Treatment Modality for Depression. Psychiatr. Clin. N. Am..

[7] Vieira W.F., Gersten M., Caldieraro M.A.K., Cassano P. (2023). Photobiomodulation for Major Depressive Disorder: Linking Transcranial Infrared Light, Biophotons and Oxidative Stress. Harv. Rev. Psychiatry.

[8] Alberti A., Vieira W.F., Coelho D.R.A., Fernandes Martins D. (2024). A Grant Report: Examining the Efficacy of Remote Photobiomodulation Therapy in Adolescents with Major Depressive Disorder. Photonics.

[9] Coelho D.R.A., Puerto A.M.H., Vieira W.F., Lohmann C.A., Shahab M.H., Gersten M.B., Vahedifard F., McEachern K.M., Clancy J.A., Cassano P. (2024). Transcranial Photobiomodulation for Executive Function in Bipolar Disorder (TPEB): Study Protocol. Photonics.

[10] Gaggi N.L., Collins K.A., Gonzalez-Castillo J., Hurtado A.M., Castellanos F.X., Osorio R., Cassano P., Iosifescu D.V. (2024). Transcranial Photobiomodulation Increases Intrinsic Brain Activity within Irradiated Areas in Early Alzheimer’s Disease: Potential Link with Cerebral Metabolism. Brain Stimul..

[11] Coelho D.R.A., Gersten M., Jimenez A.S., Fregni F., Cassano P., Vieira W.F. (2024). Treating Neuropathic Pain and Comorbid Affective Disorders: Preclinical and Clinical Evidence. Pain. Pract..

[12] Liebert A., Bicknell B., Laakso E.-L., Heller G., Jalilitabaei P., Tilley S., Mitrofanis J., Kiat H. (2021). Improvements in Clinical Signs of Parkinson’s Disease Using Photobiomodulation: A Prospective Proof-of-Concept Study. BMC Neurol..

[13] Coelho D.R.A., Renet C., López-Rodríguez S., Cassano P., Vieira W.F. (2024). Transcranial Photobiomodulation for Neurodevelopmental Disorders: A Narrative Review. Photochem. Photobiol. Sci..

[14] Vieira W.F., Coelho D.R.A., Gersten M., Puerto A.M.H., Kalli S., Gonzalez-Garibay G., McEachern K., Clancy J.A., Skotko B.G., Abbeduto L. (2024). TransPhoM-DS Study Grant Report: Rationale and Protocol for Investigating the Efficacy of Low-Power Transcranial Photobiomodulation on Language, Executive Function, Attention, and Memory in Down Syndrome. Photonics.

[15] Coelho D.R.A., Salvi J.D., Vieira W.F., Cassano P. (2024). Inflammation in Obsessive-Compulsive Disorder: A Literature Review and Hypothesis-Based Potential of Transcranial Photobiomodulation. J. Neurosci. Res..

[16] Gaggi N.L., Roy N.L., Song X., Peterson A.L., Iosifescu D.V., Diaz-Arrastia R., Cassano P., Kim J.J. (2024). Transcranial Photobiomodulation and Chronic Traumatic Brain Injury. Photonics.

[17] Cassano P., Cusin C., Mischoulon D., Hamblin M.R., De Taboada L., Pisoni A., Chang T., Yeung A., Ionescu D.F., Petrie S.R. (2015). Near-Infrared Transcranial Radiation for Major Depressive Disorder: Proof of Concept Study. Psychiatry J..

[18] Cassano P., Petrie S.R., Mischoulon D., Cusin C., Katnani H., Yeung A., De Taboada L., Archibald A., Bui E., Baer L. (2018). Transcranial Photobiomodulation for the Treatment of Major Depressive Disorder. The ELATED-2 Pilot Trial. Photomed. Laser Surg..

[19] Weerasekera A., Coelho D.R.A., Ratai E.-M., Collins K.A., Puerto A.M.H., De Taboada L., Gersten M.B., Clancy J.A., Hoptman M.J., Irvin M.K. (2024). Dose-Dependent Effects of Transcranial Photobiomodulation on Brain Temperature in Patients with Major Depressive Disorder: A Spectroscopy Study. Lasers Med. Sci..

[20] Bustillo J.R. (2013). Use of Proton Magnetic Resonance Spectroscopy in the Treatment of Psychiatric Disorders: A Critical Update. Dialogues Clin. Neurosci..

[21] Schuff N., Meyerhoff D.J., Mueller S., Chao L., Sacrey D.T., Laxer K., Weiner M.W. (2006). N-Acetylaspartate as a Marker of Neuronal Injury in Neurodegenerative Disease. Adv. Exp. Med. Biol..

[22] Pajares S., Arias A., García-Villoria J., Briones P., Ribes A. (2013). Role of Creatine as Biomarker of Mitochondrial Diseases. Mol. Genet. Metab..

[23] Michel V., Yuan Z., Ramsubir S., Bakovic M. (2006). Choline Transport for Phospholipid Synthesis. Exp. Biol. Med..

[24] Haris M., Cai K., Singh A., Hariharan H., Reddy R. (2011). In Vivo Mapping of Brain Myo-Inositol. Neuroimage.

[25] Şendur İ., Kalkan Oğuzhanoğlu N., Sözeri Varma G. (2020). Study on Dorsolateral Prefrontal Cortex Neurochemical Metabolite Levels of Patients with Major Depression Using H-MRS Technique. Turk. Psikiyatr. Derg..

[26] Xie X., Shi Y., Ma L., Yang W., Pu J., Shen Y., Liu Y., Zhang H., Lv F., Hu L. (2023). Altered Neurometabolite Levels in the Brains of Patients with Depression: A Systematic Analysis of Magnetic Resonance Spectroscopy Studies. J. Affect. Disord..

[27] Iosifescu D.V., Collins K.A., Hurtado-Puerto A., Irvin M.K., Clancy J.A., Sparpana A.M., Sullivan E.F., Parincu Z., Ratai E.-M., Funes C.J. (2023). Grant Report on the Transcranial near Infrared Radiation and Cerebral Blood Flow in Depression (TRIADE) Study. Photonics.

[28] Bottomley P.A. (1987). Spatial localization in NMR spectroscopy in vivo. Ann. N. Y. Acad. Sci..

[29] Harris A.D., Puts N.A.J., Edden R.A.E. (2015). Tissue Correction for GABA-Edited MRS: Considerations of Voxel Composition, Tissue Segmentation, and Tissue Relaxations. J. Magn. Reson. Imaging.

[30] Penny W., Friston K., Ashburner J., Kiebel S., Nichols T. (2007). Statistical Parametric Mapping: The Analysis of Functional Brain Images.

[31] Gasparovic C., Song T., Devier D., Bockholt H.J., Caprihan A., Mullins P.G., Posse S., Jung R.E., Morrison L.A. (2006). Use of Tissue Water as a Concentration Reference for Proton Spectroscopic Imaging. Magn. Reson. Med..

[32] Träber F., Block W., Lamerichs R., Gieseke J., Schild H.H. (2004). 1H Metabolite Relaxation Times at 3.0 Tesla: Measurements of T1 and T2 Values in Normal Brain and Determination of Regional Differences in Transverse Relaxation. J. Magn. Reson. Imaging.

[33] Wansapura J.P., Holland S.K., Dunn R.S., Ball W.S. (1999). NMR Relaxation Times in the Human Brain at 3.0 Tesla. J. Magn. Reson. Imaging.

[34] Davidson J., Turnbull C.D., Strickland R., Miller R., Graves K. (1986). The Montgomery-Asberg Depression Scale: Reliability and Validity. Acta Psychiatr. Scand..

[35] Pedrelli P., Blais M.A., Alpert J.E., Shelton R.C., Walker R.S.W., Fava M. (2014). Reliability and Validity of the Symptoms of Depression Questionnaire (SDQ). CNS Spectr..

[36] R Core Team (2024). R: A Language and Environment for Statistical Computing.

[37] Bates D., Mächler M., Bolker B., Walker S. (2015). Fitting Linear Mixed-Effects Models Using lme4. J. Stat. Softw..

[38] Moffett J.R., Ross B., Arun P., Madhavarao C.N., Namboodiri M.A.A. (2007). N-Acetylaspartate in the CNS: From Neurodiagnostics to Neurobiology. Prog. Neurobiol..

[39] Saccaro L.F., Tassone M., Tozzi F., Rutigliano G. (2024). Proton Magnetic Resonance Spectroscopy of *N*-Acetyl Aspartate in First Depressive Episode and Chronic Major Depressive Disorder: A Systematic Review and Meta-Analysis. J. Affect. Disord..

[40] Gonsalves M.A., White T.L., Barredo J., Fukuda A.M., Joyce H.E., Harris A.D., Carpenter L.L. (2022). Repetitive Transcranial Magnetic Stimulation-Associated Changes in Neocortical Metabolites in Major Depression: A Systematic Review. Neuroimage Clin..

[41] Naeser M.A., Martin P.I., Ho M.D., Krengel M.H., Bogdanova Y., Knight J.A., Hamblin M.R., Fedoruk A.E., Poole L.G., Cheng C. (2023). Transcranial Photobiomodulation Treatment: Significant Improvements in Four Ex-Football Players with Possible Chronic Traumatic Encephalopathy. J. Alzheimers Dis. Rep..

[42] Roy P., Tomassoni D., Nittari G., Traini E., Amenta F. (2022). Effects of Choline Containing Phospholipids on the Neurovascular Unit: A Review. Front. Cell Neurosci..

[43] Riley C.A., Renshaw P.F. (2018). Brain Choline in Major Depression: A Review of the Literature. Psychiatry Res. Neuroimaging.

[44] Yang X.-R., Langevin L.M., Jaworska N., Kirton A., Lebel R.M., Harris A.D., Jasaui Y., Wilkes T.C., Sembo M., Swansburg R. (2016). Proton Spectroscopy Study of the Dorsolateral Prefrontal Cortex in Youth with Familial Depression. Psychiatry Clin. Neurosci..

[45] Kreider R.B., Stout J.R. (2021). Creatine in Health and Disease. Nutrients.

[46] Meftahi G.H., Hatef B., Pirzad Jahromi G. (2023). Creatine Activity as a Neuromodulator in the Central Nervous System. Arch. Razi Inst..

[47] Faulkner P., Paioni S.L., Kozhuharova P., Orlov N., Lythgoe D.J., Daniju Y., Morgenroth E., Barker H., Allen P. (2021). Relationship between Depression, Prefrontal Creatine and Grey Matter Volume. J. Psychopharmacol..

[48] Guo Z.-L., Liu T.C.-Y. (2022). Quantitative and Integrative Photobiomodulation. Photobiomodul. Photomed. Laser Surg..

